# Exosome: The Regulator of the Immune System in Sepsis

**DOI:** 10.3389/fphar.2021.671164

**Published:** 2021-04-28

**Authors:** Peng Qiu, Jing Zhou, Jin Zhang, Youjing Dong, Yang Liu

**Affiliations:** ^1^ Department of Anesthesiology, Shengjing Hospital of China Medical University, Shenyang, China; ^2^ Department of Oncology, Shengjing Hospital of China Medical University, Shenyang, China

**Keywords:** exosome, extracellular vesicle, sepsis, immune, antigen presentation, innate immune, adaptive immune

## Abstract

Sepsis is a syndrome comprised of a series of life-threatening organ dysfunctions caused by a maladjusted body response to infection with no effective treatment. There is growing evidence that the immune system plays a core role in sepsis. Pathogens cause abnormal host immune response and eventually lead to immunosuppression, which is an important cause of death in patients with sepsis. Exosomes are vesicles derived from double invagination of plasma membrane, associating with immune responses closely. The cargos delivered by exosomes into recipient cells, especially immune cells, effectively alter their response and functions in sepsis. In this review, we focus on the effects and mechanisms of exosomes on multiple immune cells, as well as the role of immune cell-derived exosomes in sepsis. This is helpful for us to have an in-depth understanding of the mechanism of immune disorders in sepsis. Exosomes is also expected to become a novel target and therapeutic approach for sepsis.

## Introduction

Sepsis is a syndrome of multiple life-threatening organ dysfunction caused by the dysregulated host response to infection (Singer et al., 2016). With the progress of intensive care management and goal-directed interventions, early mortality of sepsis has diminished (Delano and Ward, 2016; Steinhagen et al., 2020). However, persistent inflammation, immunosuppression and catabolism syndrome (PICS) in later phase of sepsis remains unsolved, which is the main cause of death in septic patients (Venet and Monneret, 2018). At present, increasing evidence supports the core role of the immune system in sepsis (van der Poll et al., 2017; Rubio et al., 2019). In sepsis, the immune response initiated by invading pathogens failed to return homeostasis, which eventually leads to a pathological syndrome characterized by persistent excessive inflammation and immunosuppression (van der Poll et al., 2017). Therefore, understanding the complex mechanism of immune imbalance in sepsis and the application of targeted immunotherapy has become a research hotspot in the field of sepsis. Abnormal activation, massive apoptosis, phenotypic, and functional changes of immune cells are the pathological basis of immune disorders, especially immunosuppression in sepsis (Hotchkiss et al., 2013). The use of cytokines such as interleukin-7 (IL-7), interleukin-15 (IL-15), granulocyte-macrophage colony-stimulating factor (GM-CSF) and co-inhibitory molecules blockade involving anti-programmed cell death receptor-1 (anti-PD-1) and anti-B and T lymphocyte attenuator (anti-BTLA) have been proven to reduce the mortality of sustained sepsis (Leentjens et al., 2013; Hutchins et al., 2014; Delano and Ward, 2016; van der Poll et al., 2017; Venet and Monneret, 2018; Steinhagen et al., 2020).

**FIGURE 1:**
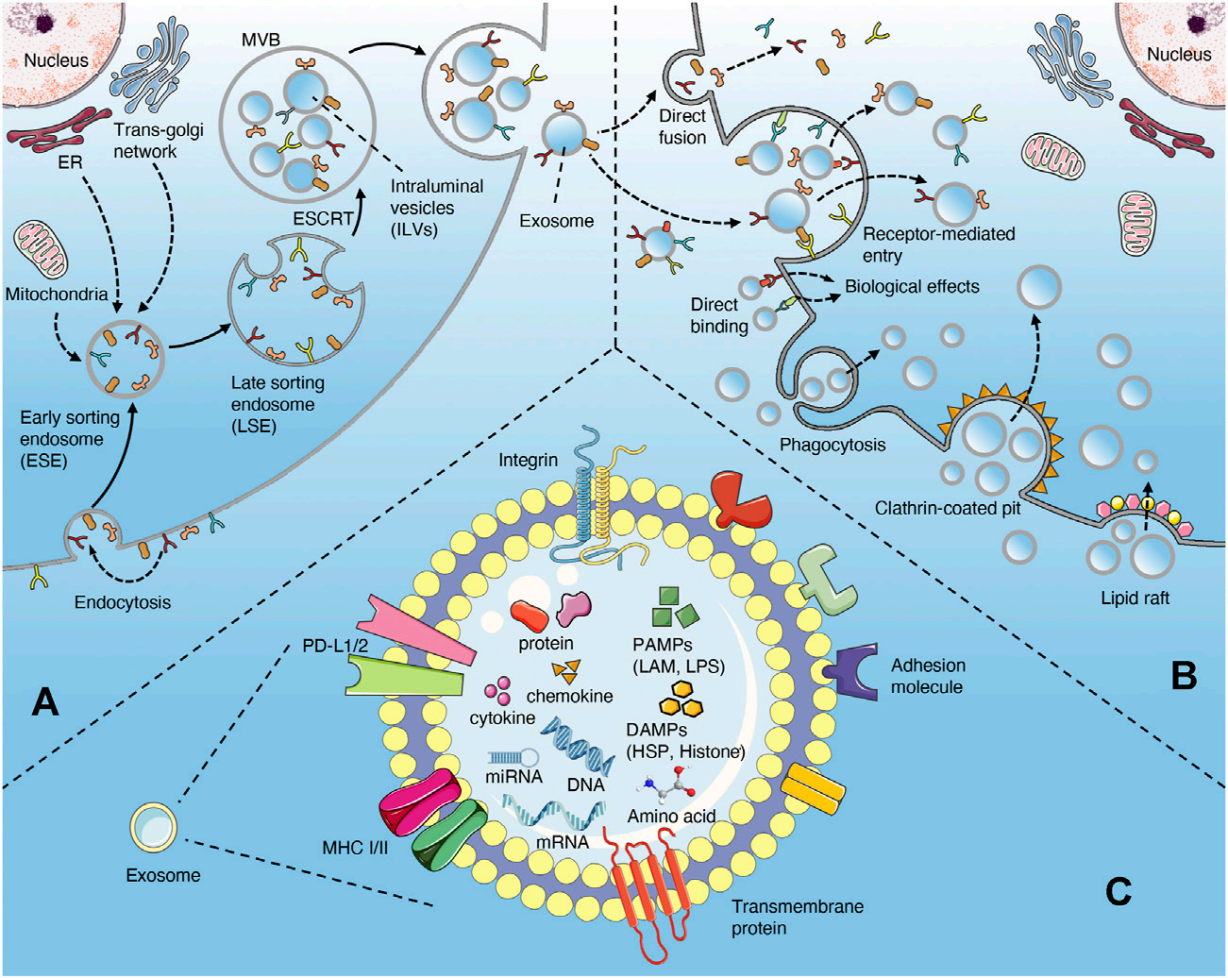
Biogenesis and uptake of exosomes **(A)** the exosomes originate from the invagination of the plasma membrane, followed by the formation of ESE, LSE, MVB (contain ILVs), and ultimately be released through the fusion of MVB with the plasma membrane **(B)** the uptake of exosomes may involve directly fusion, receptor-mediated endocytosis, phagocytosis, clathrin-coated pits and lipid raft. In addition, exosomes can interact with cells *via* directly binding way **(C)** the contents of exosomes in sepsis include proteins, nucleic acids, cytokines, chemokines, PAMPs, and DAMPs.

Exosomes are a subset of extracellular vesicles (EVs), with a diameter of 40–160 nm, derived from endosome (Kalluri and LeBleu, 2020). The biogenesis of exosomes includes the double invagination of plasma membrane and the formation of multivesicular body (MVB) which containing intraluminal vesicles (ILVs). ILVs are subsequently released into extracellular space according to MVB fusion with the plasma membrane and exocytosis, which are named exosomes ultimately (Robbins and Morelli, 2014; Kalluri and LeBleu, 2020). Exosomes are enriched in a variety of cell surface proteins, intracellular proteins, amino acids, nucleic acids (DNA and RNA), lipids and metabolites, which can mediate intercellular communication and affect the biological function of recipient cells (Kalluri and LeBleu, 2020) (Figure 1C). Exosomes have been proved to play an important role in multiple diseases (Pegtel and Gould, 2019), including tumor (Hoshino et al., 2015), infection (Rialdi et al., 2017), inflammation (Choi et al., 2020), cardiovascular diseases (Barile et al., 2017) and autoimmune diseases (Majer et al., 2019). The effects of exosomes on immune system involving antigen presentation, immune cells maturation, differentiation and activation, as well as their applications as drug carriers for immunotherapy have been widely studied (Chaput and Théry, 2010; Bobrie et al., 2011; Robbins and Morelli, 2014; Robbins et al., 2016). In addition, exosomes have also become an important target and approach for the treatment of sepsis (Terrasini and Lionetti, 2017; Wu et al., 2017; Raeven et al., 2018; Murao et al., 2020). This review focuses on the immunomodulatory effect of exosomes in sepsis. We summarized the effects (activation or inhibition) and underlying mechanisms of exosomes of different origins (non-immune and immune cells) and components on the immune system.

## Immunopathology of Sepsis

The pathological process of sepsis is mainly manifested by excessive inflammatory response and consequent immunosuppression (van der Poll et al., 2017). When infection occurs, the pathogen-associated molecular patterns (PAMPs) of invading pathogens are recognized by innate immune cells through various pattern recognition receptors (PRRs), involving toll-like receptors (TLRs), nucleotide oligomerization domain-like receptors (NLRs), C-type lectin receptors (CLRs), and RigI-helicases (Takeuchi and Akira, 2010; Kumar et al., 2011). In general, the innate immune system eliminates pathogens and restores homeostasis through a variety of pro-inflammatory reactions. Once the pathogen prevails and is not effectively cleared, the host immune response becomes unbalanced, and eventually leading to sepsis (van der Poll et al., 2017). Excessive inflammatory responses are mobilized against the threat of pathogens, which can induce abnormal activation of the complement system and coagulation system and vascular endothelial dysfunction (van der Poll et al., 2017). Complements C3a and C5a have powerful pro-inflammatory effects, including recruitment and activation of leukocytes and platelets (Merle et al., 2015), which adhere to the surface of endothelial cells and lead to barrier dysfunction through excessive inflammatory response. Intense activation of the coagulation system can lead to disseminated intravascular coagulation (DIC). In addition, excessive inflammation trigger cell injury and the release of damage-associated molecular patterns (DAMPs), which result in further activation of the innate immune system and inflammation outbreak, eventually leading to organ damage and dysfunction (van der Poll et al., 2017).

Persistent excessive inflammation triggers extensive apoptosis in immune cells (especially lymphocytes and dendritic cells) (Boomer et al., 2011; Hotchkiss et al., 2013), while delays apoptosis of neutrophils (Tamayo et al., 2012). However, these neutrophils have lower bactericidal functions and decreased cytokine production (Tamayo et al., 2012). The expression of programmed cell death 1 (PD1) of CD4+ T cells and the proportion of regulatory T (Treg) cells increase, resulting in the inhibition of effector T cell function (Leentjens et al., 2013). Monocytes and macrophages have a reduced ability to release pro-inflammatory cytokines under stimulation such as LPS, which also known as “immuno-paralysis” (Hotchkiss et al., 2013). These factors contribute to severe immunosuppression in sepsis, especially in the later stages, leading to an increased chance of secondary infection (van der Poll et al., 2017). In summary, the immune mechanism is the core throughout the occurrence and progression of sepsis. Immunotherapy will become the key to the treatment of sepsis.

## Biogenesis and Uptake of Exosomes

The biogenesis of exosomes mainly depends on the endosomal sorting complexes required for transport (ESCRT) mechanism (Robbins and Morelli, 2014; Kalluri and LeBleu, 2020) (Figure 1A). Extracellular components, including proteins, lipids, metabolites can enter cells together with cell surface proteins through endocytosis or plasma membrane invagination (Kalluri and LeBleu, 2020). With the participation of endoplasmic reticulum (ER), trans-Golgi network (TGN) and mitochondrial constituents, this membrane budding process drives the formation of early-sorting endosome (ESE) and gradually matures into late-sorting endosome (LSE)(Kalluri, 2016; Hessvik and Llorente, 2018; Mathieu et al., 2019). LSE eventually transforms into multivesicular body (MVB) which containing Intraluminal vesicles (ILVs) through ESCRT-mediated secondary invagination of the plasma membrane (Raiborg and Stenmark, 2009; Hurley and Hanson, 2010). After the fusion of MVB with plasma membrane, these ILVs are released out of the cell *via* exocytosis and become exosomes (Robbins and Morelli, 2014; Kalluri and LeBleu, 2020). Some of MVBs can also be degraded through autophagy or lysosomal pathway (Robbins and Morelli, 2014; Kalluri and LeBleu, 2020). In addition, certain proteins, such as PLP, are also sorted into ILVs through a machinery independently of ESCRT (Stuffers et al., 2009). The uptake of exosomes by target cells may involve plasma membrane fusion, receptor-mediated endocytosis (i.e. ICAM1 binding with LFA1), clathrin-coated pits, lipid raft, caveolae and phagocytosis (such as PS binding with its ligands MFGE8 and Tim1/4). In addition, exosomes can bind to cells directly *via* surface receptor or ligand proteins (Figure 1B) (Chaput and Théry, 2010; Kalluri and LeBleu, 2020). Exosomes release bioactive contents to recipient cells after internalization, or directly induce the activation of intracellular signaling pathways through ligand-receptor binding (Kalluri and LeBleu, 2020).

## Exosomes and Immune System

Exosomes derived from both immune and non-immune cells play an important role in immune regulation, which may promote the pathology of multiple diseases through mediating immune stimulation or suppression (Robbins and Morelli, 2014). The immune regulation of exosomes may be due to the antigenic peptides transfer and presentation, the transmission of cGAS-STING signals induced by DNA in recipient cells, the regulation of gene expression by exosome miRNA, and the induction of different signaling by exosome surface ligands (such as PD-L1 and FasL) (Kalluri and LeBleu, 2020). Interestingly, recent study has revealed that exosomes also provide protection similar to innate immunity by neutralizing bacterial toxins actively (Keller et al., 2020).

### Exosomes Participate in Antigen Presentation

**FIGURE 2:**
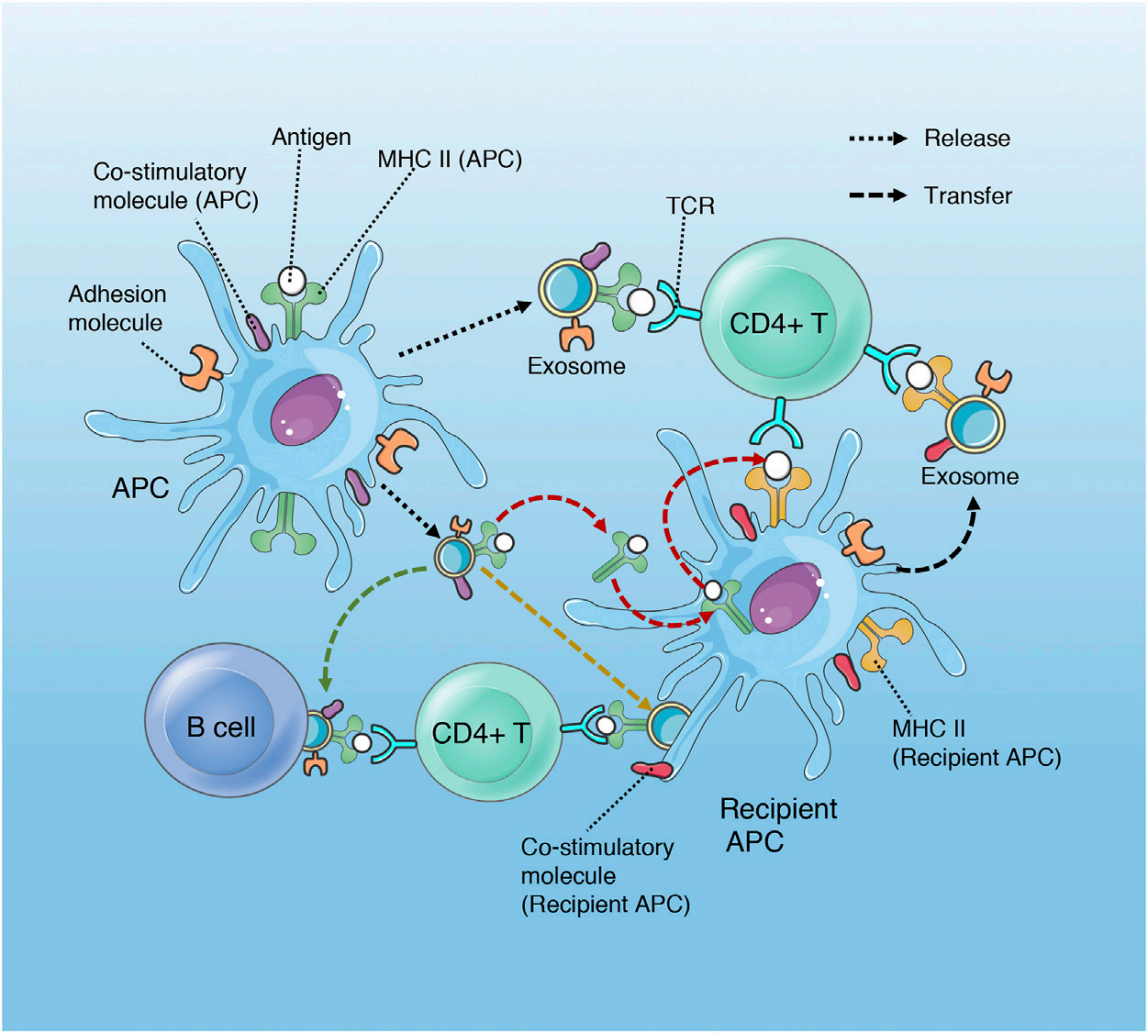
Role of exosomes in antigen presentation. Exosomes released by APCs (DCs) contain MHC II- antigen peptide complexes (MHC II-p) and can present antigen to CD4+T cell directly, deliver antigen to MHC II of recipient APC (red arrow), present antigen *via* cross-dressing (orange arrow) and transport MHC II-p to B cell (green arrow). Although only MHC II and CD4+ T cell are shown, exosomal MHC I has a similar process in the regulation of CD8+ T cells.

Traditional antigen presentation requires antigen presenting cells (APCs) to process antigenic peptides and form MHC-peptide complexes, which subsequently promote T cell activation and proliferation *via* binding to the T cell receptor (TCR) with the synergism of co-stimulatory molecules. However, exosomes can complete antigen presentation without the interaction between APCs and T cells, or even without the reprocessing of MHC-peptide complex by the recipient APCs, which promote the efficiency of presentation and favor the host to initiate immuno-defense against invading pathogens more quickly. Exosomes participate in antigen presentation mainly through the following three mechanisms (Segura et al., 2005; Chaput and Théry, 2010; Robbins and Morelli, 2014; Tung et al., 2018) (Figure 2). 1) direct presentation: exosomes derived from the professional APCs (that is, dendritic cells (DCs), which carry the MHC-peptide complexes, co-stimulatory and adhesion molecules, bind with T cells directly. 2) indirect presentation: exosomes transfer their antigenic peptide to the MHC molecules of the recipient APCs. After loading with the exosome-derived peptide, the recipient APCs then present the MHC-peptide complexes to T cells. 3) cross-dressing: the captured exosomes that are retained on the APCs surface present their MHC- peptide complexes directly to T cells, although the co-stimulatory molecules are provided by the APCs.

In addition, dendritic cells may transfer the ability of activating T cells to non-professional antigen presenting cells (B cell and macrophage) through exosome-mediated antigen presentation (Segura et al., 2005; Robbins and Morelli, 2014).

### Active Immuno-Defense of Exosomes

Bacteria disrupt the plasma membrane of host cells by releasing pore-forming toxins and cause cell death during infection (Dal Peraro and van der Goot, 2016; Seilie and Bubeck Wardenburg, 2017; Spaan et al., 2017). Matthew et al. found that cells exposed to bacteria release exosomes that containing ADAM10 in an ATG-dependent manner, which can neutralize α-toxin (a pore-forming toxin) produced by methicillin-resistant Staphylococcus aureus (MRSA) and protect host cells from death (Keller et al., 2020). These exosomes serve as decoy to bind bacterial toxins and play the role of scavenger similar to innate immune cells (Keller et al., 2020). This finding indicates the active immuno-defensive role of exosomes during infection, except for mediating intercellular communication.

## Effects of Exosomes on Immune Cells in Sepsis

The immuno-modulatory effect of exosomes depends on multiple factors involving type of disease, cellular sources and the cargos transmitted to the recipient cells (Robbins and Morelli, 2014; Kalluri and LeBleu, 2020). The immune regulation of exosomes in sepsis also exhibit pleiotropy and complexity. Exosomes from distinct cellular sources or the same source with different cargos can activate or suppress immune cells. The role of endogenous exosomes is both beneficial and harmful, while exogenous exosomes are often used as drug carriers for sepsis immunotherapy (Terrasini and Lionetti, 2017; Wu et al., 2017; Raeven et al., 2018; Murao et al., 2020).

### Immunomodulatory Effect of Exosomes Derived From Non-immune Cells in Sepsis

#### Serum and Platelets

**FIGURE 3:**
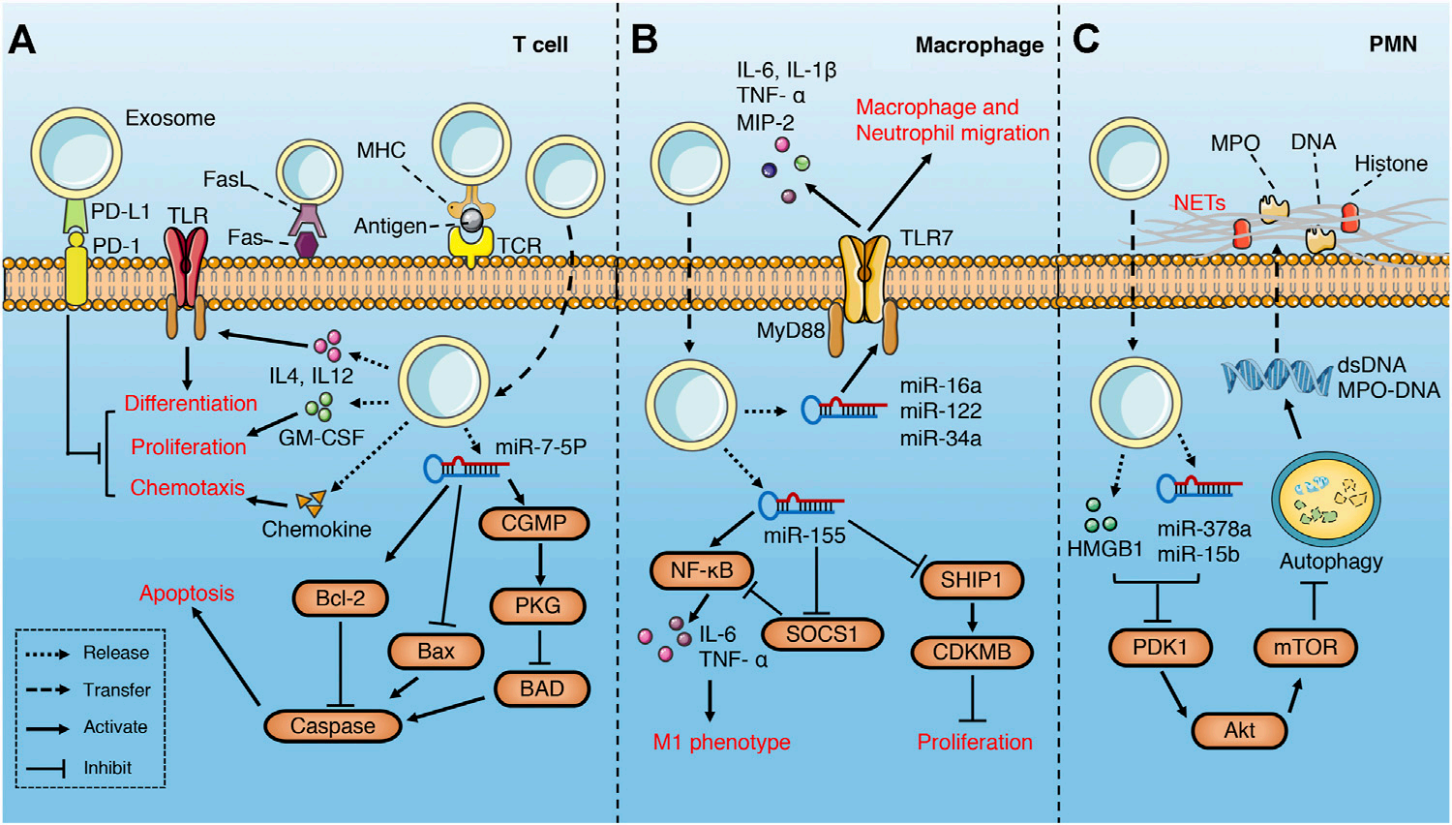
Role of exosomes derived from serum in sepsis. **(A)** serum exosomes can promote differentiation, proliferation and chemotaxis of T cell *via* pro-inflammatory cytokines, GM-CSF and chemokines separately, while play the opposite role through PD1/PDL1 pathway. In addition, exosomes may attenuate T cell apoptosis through miR-7-5p-mediated inhibition of caspase. However, exosomes may also induce T cell apoptosis *via* FasL/Fas signaling pathway. **(B)** serum exosomes promote macrophage migration, proliferation and M1 polarization through multiple miRNAs-mediated signaling pathways. **(C)** Platelet exosomes induce excessive NETs formation through Akt/mTOR autophagy pathway.

At present, a number of studies have proved that abundant exosomes exist in the plasma of septic patients and animal models (Table 1), which affect the function of a variety of immune cells (Figure 3), including T lymphocytes (Alexander et al., 2015; Deng et al., 2019; Gao et al., 2019), macrophages (Xu et al., 2018; Jiang et al., 2019), neuroglia cells (Li et al., 2018a) and neutrophils (Jiao et al., 2020).

**TABLE 1:** Immunomodulatory effect of exosomes derived from non-immune cells in sepsis.

Donor cell/tissue	Contents	Transfer pathway	Target cell	Signaling pathway/Protein	Immuno-effect	Immune outcome	References
Serum	Cytokines(IL, TNF-α, IFN-γ), chemokine, GM-CSF	TCR	T cell	TLR4 (+)	T cell differentiation, proliferation and chemotaxis ↑	Activation	Gao et al. (2019)
Serum	Has-miR-7-5p	N/A	T cell	cGMP-PKG (+)	T cell apoptosis ↓ (BAD ↓, caspase3 ↓, bax ↓, Bcl2 ↑)	Activation	Deng et al. (2019)
Serum	miR-155	N/A	Macrophage	SHIP1-CDKN1B (−) SOCS1 (−) NF-κb (+)	Macrophage proliferation ↑, M1 ↑, TNF-α ↑, IL-6 ↑	Activation	Jiang et al. (2019)
Serum	EVs^a^: miR-16a, miR-122, miR-34a	N/A	Macrophage neutrophil	TLR7-MyD88 (+)	Macrophage and neutrophil migration ↑, MIP-2 ↑, IL-6 ↑, IL-1β↑, TNF-α ↑, FB ↑, C3 ↑	Activation	Xu et al. (2018)
Serum	miR-21, miR-125a, miR-146a, miR-155	N/A	Microglia Astrocyte	N/A	Inflammation ↑	Activation	Li et al. (2018a)
Serum	miR-125b, miR-27a, mRNA	N/A	N/A	N/A	MPO ↑, FOXM1 ↑, inflammation ↑	N/A	Real et al. (2018)
Serum	EVs^a^: Integrin β2, PDL-1/2	Integrin β2	Lymphocyte	PD-1/PDL-1/2 (+)	Lymphocyte numbers ↓	Suppression	Kawamoto et al. (2019)
Serum	MHC-II, CD11b	N/A	N/A	Fas/FasL (+)	Inflammation ↓	Suppression	Kim et al. (2007)
Platelet	HMGB1, miR-15b-5p, miR-378a-3p	IKK	Neutrophil	Akt-mTOR (+)	dsDNA + MPO-DNA complex ↑, NETs ↑	Activation	Jiao et al. (2020)
BMSC	N/A	N/A	Macrophage	HIF-1α-glycolysis (−)	M2 ↑, M1 ↓	Suppression	Deng et al. (2020)
BMSC	miR-21	IL-1β	Macrophage	PDCD4 (−)	M2 ↑, M1 ↓	Suppression	Yao et al. (2021)
AMSC	miR-34a-5p, miR-146a-5p, miR-21	TNF-α, IFN-γ	Macrophage	Notch1 (−), IRAK1-TRAF6 (−), Sirp-β1 (late) (−), STAT3 (early) (+), MEK/ERK1/2 (+)	M2 ↑, M1 ↓, M1 ↑ (early)	Suppression	Domenis et al. (2018)
BMSC	miR-223	N/A	Cardiac myocyte	Sema3A (−), STAT3 (−)	Myocardial protection	Suppression	Wang et al. (2015c)
MSC	N/A	N/A	THP1 (direct), CD4+T cell (indirect)	TLR-MyD88-NF-κB (+)	M2 Phenotype↑, Treg ↑, IL-10 ↑	Suppression	Zhang et al. (2014)
AMSC	N/A	N/A	T cell	NF-κB (?)	CD4^+^T and CD8^+^T cell proliferation ↓, T cell differentiation ↓, IFN-γ ↓	Suppression	Blazquez et al. (2014)
MSC	N/A	N/A	PBMC (PMN, lymphocyte)	N/A	TGF-β↑, IL-4↑, TNF-α ↓, IL-1β ↓, IFN-γ ↓, IL-17 ↓Th1 ↓, Th2 ↑, Th17 ↓, Treg ↑, CTP4 ↑, PBMC and CD3^+^T cell apoptosis↑	Suppression	Chen et al. (2016)
BMSC	miR-146a	N/A	Macrophage	IRAK1 (-), TRAF6 (-), IRF5 (-)	M2 ↑, TNF-α ↓, IL-10 ↑	Suppression	Song et al. (2017)
MSC	MV^a^: KGF mRNA	KGF	Alveolar epithelial cell (direct) Macrophage (indirect) Neutrophil (indirect)	N/A	TNF-α↓, IL-10↑, MIP2↓, neutrophil influx ↓	Suppression	Zhu et al. (2014)
MSC	MV^a^: CD44, mRNA (KGF, COX2, IL-10, mitochondria)	CD44-L- selectin/osteopontin, TLR-2/3/4	Alveolar epithelial type II cell, monocyte/Macrophage	COX2-PGE2 (+)	ATP (Alveolar epithelial cell) ↑, phagocytosis (monocyte) ↑, M2 ↑, M1 ↓, PGE2 ↑, IL-10 ↑, TNF-α ↓	Suppression	Monsel et al. (2015)
BMSC	EVs^a^: CD44, mRNA (mitochondria)	CD44-L- selectin/osteopontin	Macrophage	OXPHOS (+)	Phagocytosis ↑, M2 ↑, M1 ↓, TNF-α ↓, IL-8 ↓	Suppression	Morrison et al. (2017)
MSC	N/A	N/A	Microglia Astrocyte	N/A	Microglia proliferation ↓ (Ibα-1 ↓, CD68 ↓) Astrocyte proliferation ↓ (GFAP ↓)	Suppression	Drommelschmidt et al. (2017)
MSC	EVs^a^: TGF-β, IL-10	N/A	Motor neuron	N/A	Inflammation ↓	Suppression	Rajan et al. (2016)
MSC	miR-181c	N/A	Macrophage Neutrophil	TLR4- NF-κB (-)	Macrophage and neutrophil infiltration ↓, IL-1β ↓, TNF-α↓	Suppression	Li et al. (2016)
MSC	N/A	TLR	DC (direct) T cell (indirect)	N/A	IL-6 ↓, IL-10 ↑, TGF-β ↑, TolDC ↑, treg ↑	Suppression	Shahir et al. (2020)
EC	HSPA12B	N/A	Macrophage	PI3K/AKT (+) NF-κB (-)	Macrophage infiltration ↓, monocyte/Macrophage activation ↓, IL-1β ↓, TNF-α ↓, IL-10 ↑	Suppression	Tu et al. (2020)
HMEC-1	MV^a^: EMP	Sodium-proton exchanges, Intact cytoskeleton	PDC (plasmacytoid dendritic cell)	N/A	IL-6 ↑, IL-8 ↑, co-stimulatory molecules (CD80/86/40) ↑, HLA-DR ↑, CD83 ↑, CCR7 ↑, TNF-α ↑, IFN-γ ↑, Th1 ↑	Activation	Angelot et al. (2009)
HUVEC	EVs^a^: miR-10a, miR-12b, miR-181b	N/A	Monocyte (THP-1)	Nucleus: NF-κB (−), IRF5 (−) Cytoplasm: IRAK4 (−), TAK1/MAP3K7 (−), βTRC-NF-κB (−)	Pro-inflammatory response ↓, immunomodulatory phenotype ↑, M2 ↑, M1 ↓	Suppression	Njock et al. (2015)
EC	EVs^a^: miR-155	N/A	Monocyte (THP-1)	N/A	OX-LDL: M1 ↑, M2 ↓, KLF2: M2 ↑, M1 ↓	Activation/Suppression	He et al. (2018)
Alveolar epithelial type II cell	miR-146a	N/A	Alveolar macrophage	TLR4-IRAK1-TRAF6-NF-κB (−)	IL-6 ↓, IL-8 ↓, IL-1β ↓, TNF-α ↓	Suppression	Zheng et al. (2020)
TEC	miR-19b-3p	N/A	Macrophage	SOCS1 (−)	M1 ↑	Activation	Lv et al. (2019)
Lung epithelial cell	EVs^a^: Caspase-3	N/A	Alveolar macrophage Neutrophil	ROCK1 (+)	MIP-2 ↑, pro-inflammatory cytokines ↑, macrophage and neutrophil infiltration ↑	Activation	Moon et al. (2015)
Lung epithelial cell	EVs^a^: miR-17, miR-221	N/A	Macrophage	PTEN (-), c-fos-rab11 (+)	Integrin β1 expression and circulation ↑, Macrophage recruitment and migration ↑	Activation	Lee et al. (2017)
Lung epithelial cell	EVs^a^: miR-320a, miR-221	N/A	Macrophage	MMP9 (+), NF-κb (+)	Macrophage migration and activation ↑, IL-1β ↑, TNF-α ↑	Activation	Lee et al. (2016)
CPE	EVs^a^: miR-146a, miR-155	Cross the ependymal cell layer	Microglia Astrocyte	Target mRNA (−)	Inflammatory gene ↑	Activation	Balusu et al. (2016)
IEC	EVs^a^: miRNA	N/A	IEC	N/A	TNF-α ↓, IL-17a ↓	Suppression	Appiah et al. (2020)
IEC	PGE2	N/A	Hepatocyte (direct) NKT cell (indirect) CD4+T cell (indirect)	EP2/4-CAMP-PKA (+)	Hepatocyte apoptosis ↓, NKT cell activation ↓, CD4^+^T cell activation ↓, IFN-γ ↓, TNF-α ↓, IL-4 ↓, IL-2 ↓	Suppression	Deng et al. (2013)
IEC Neutrophils	EVs^a^: miRNA, IL-6/8, TNF-α	N/A	IEC, Macrophage	N/A	Macrophage migration ↑, pro-inflammation ↑	Activation	Mitsuhashi et al. (2016)
Synovial fibroblasts	TNF-α (membrane form)	TNF-α ubiquitin	T cell	AKT-NF-κb (+)	T Cell apoptosis ↓ (caspase-3 ↓)	Activation	Zhang et al. (2006)

^a^ Exosomes are subsets of EVs or MVs, however, their immunomodulatory effects may exist unpredictable differences.

Gao et al. (Gao et al., 2019)found that plasma exosomes enriched with IL-12 and IL-4 effectively promote the differentiation of Th1/Th2 cells in the middle and late phase of sepsis, while the growth factor GM-CSF in the exosomes augments the proliferation of T cells through TLR4-dependent pathway. Although failed to recruit lymphocytes directly, the exosomes with chemokine enhance the migration of lymphocytes (Gao et al., 2019). All these are beneficial to the resuscitation of immunosuppressive state in the late phase of sepsis and reduce the mortality. Similarly, Deng et al. (Deng et al., 2019) demonstrated that exosomes derived from plasma of septic patients downregulate the mRNA and protein levels of pro-apoptotic gene Bad, active Caspase-3 and Bax, while upregulate that of anti-apoptotic gene Bcl-2 *via* hsa-miR-7-5p, thus inhibit apoptosis of T lymphocytes induced by lipopolysaccharide (LPS). The inhibition of Bad by hsa-miR-7-5p may be related to the activation of CGMP-PKG pathway (Deng et al., 2019). Exosomes derived from the serum of septic mice deliver miR-155 to macrophages and promote M1 polarization *via* activating NF-kB, while enhance macrophages proliferation by targeting inhibition of SHIP1 and SOCS1(Jiang et al., 2019). Similarly, miR-155 in the serum exosomes also leads to the proliferation and activation of microglia and astrocytes in LPS-treated mice, which aggravate the inflammatory response of the nervous system (Li et al., 2018a). In addition, miR-34a, miR-122 and miR-146a in plasma EVs of septic mice increase the release of pro-inflammatory cytokines (IL-6, IL-1 β, TNF- α and MIP-2) by macrophages in a TLR7-MyD88-dependent manner and promote the migration of macrophages and neutrophils, all of which activate immune system and aggravate the inflammatory response of sepsis (Xu et al., 2018).

On the contrary, plasma-derived exosomes can also lead to immunosuppression. Integrins have been shown to be involved in regulating the biological distribution of exosomes and the binding and internalization of target cells (Hoshino et al., 2013; Wang et al., 2015b). Kawamoto et al. (Kawamoto et al., 2019) found that integrins and PD-1 ligands (PDL-1 and PDL-2) enriched in plasma EVs of septic patients synergistically promote the binding of EVs and lymphocytes, and inhibit the activation of T cells through the negative signal transmitted by PD-1. Similarly, Kim et al. (Kim et al., 2007)found that plasma exosomes carrying MHC II and CD11b suppress antigen-specific immune responses partially through Fas/FasL-dependent pathways.

Platelets are the main source of circulating exosomes (Ogura et al., 2001). Platelet-derived exosomes containing HMGB1, miR-15b-5p and miR-378a-3p induce excessive neutrophil extracellular trap (NET) formation through Akt/mTOR autophagy pathway (Jiao et al., 2020), which aggravates vascular endothelial injury and coagulation dysfunction (Caudrillier et al., 2012; Leppkes et al., 2019).

#### Mesenchymal Stem Cell

**FIGURE 4:**
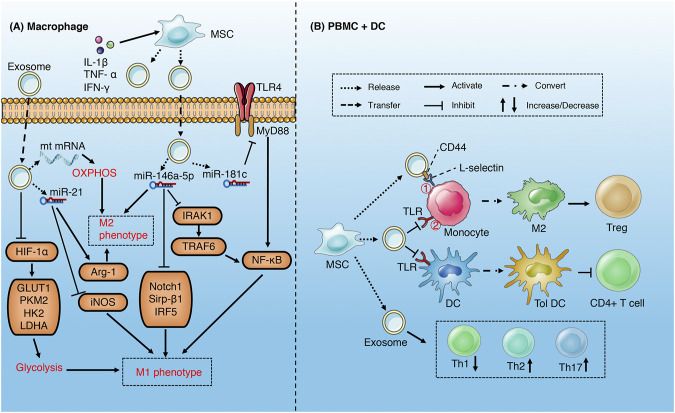
Role of exosomes derived from MSC in sepsis **(A)** MSC-derived exosomes increase M2 and decrease M1 phenotype of macrophages through metabolism reprogramming and multiple miRNAs-mediated signaling pathways **(B)** MSC-derived exosomes induce M2-like phenotype in monocytes *via* activating COX2-PGE2^①^ and inhibiting TLR/NF-KB^②^ signaling pathways, which in turn induce the expansion of Treg. MSCs-derived exosomes induce DCs into a tolerogenic population and modulate the differentiation spectrum of T cell subsets.

Mesenchymal stem cell (MSCs) can be easily isolated from tissue and expanded *in vitro*. For the pluripotency and immune activity, MSCs has become an effective treatment for many diseases including sepsis. MSCs can inhibit T cell proliferation and cytotoxicity, regulate the function of regulatory T cells, inhibit B cell proliferation, regulate the maturation, activation and antigen presentation of dendritic cells, regulate natural killer cell activation (Gao et al., 2016). MSC-derived exosomes play an anti-inflammatory role in sepsis by suppressing the immune function of monocytes/macrophages, dendritic cells, neutrophils and T cells (Table 1) (Figure 4).

Studies have demonstrated that MSCs-derived exosomes may alleviate the inflammatory injury induced by sepsis *via* reprogramming the metabolism of macrophages (Morrison et al., 2017; Deng et al., 2020). M1 macrophages acquire energy from aerobic glycolysis, while M2 macrophages obtain energy through mitochondrial oxidative phosphorylation (Zhu et al., 2015). In addition, aerobic glycolysis is an important pathway of macrophage activation and M1 polarization (Pan et al., 2020; Zhao et al., 2020). MSCs-derived exosomes suppress LPS-induced glycolysis of macrophage *via* inhibition of HIF-α and its downstream pathway and reduce M1 polarization. Correspondingly, exosomes promote oxidative phosphorylation through delivering mitochondrial mRNA to macrophages, which increases M2 polarization (Morrison et al., 2017). The effect of MSCs-derived exosomes on macrophage polarization can be enhanced by the stimulation of pro-inflammatory factors (Domenis et al., 2018; Yao et al., 2021). Under stimulation of pro-inflammatory cytokines (IL-1β, TNF-α and IFN-γ), MSCs released exosomes rich in miR-34a-5p (Domenis et al., 2018), miR-146a-5p (Song et al., 2017; Domenis et al., 2018) and miR-21 (Domenis et al., 2018; Yao et al., 2021), which promote the phenotypic transition from M1 to M2 by inhibiting Notch1, IRAK1/TRAF6, IRF5, and Sirp-β1 signaling pathways and related proteins. In addition, CD44 in exosomes can enhance the phagocytosis of macrophages and inhibit the release of TNF-α (Morrison et al., 2017). The exosomes derived from human umbilical cord mesenchymal stem cells carrying miR-181c reduce macrophage infiltration and excessive release of inflammatory factors, which is partly attributed to the inhibition of TLR4/NF-κB signaling pathway (Li et al., 2016).

MSCs-derived exosomes can induce an anti-inflammatory M2-like phenotype in monocytes *via* inhibiting TLR/NF-KB signaling pathway in sepsis, which subsequently induce the expansion of regulatory T (Treg) cells (Zhang et al., 2014; Chen et al., 2016). Similarly, EVs (exosomes) up-regulated the expression of PGE2 *via* transferring COX2 mRNA to monocytes, which may increase M2-like phenotype (Monsel et al., 2015). MSCs-derived exosomes can also regulate the proliferation and activation of T cells in different subsets, including inducing the conversion of Th1 to Th2, reducing the potential of T cells to differentiate into Th17 (Blazquez et al., 2014; Chen et al., 2016), and reducing the release of pro-inflammatory factors such as IFN- γ (Blazquez et al., 2014). MSCs-derived exosomes induce DCs into a tolerogenic DC (TolDC) population, which are capable of suppressing lymphocyte activities, increasing secretion of IL-10 and TGF-β and decreasing secretion of IL-6 (Shahir et al., 2020). In addition, MSCs-derived exosomes may also reduce septic nervous system inflammation by suppressing the proliferation of microglia and astrocytes (Rajan et al., 2016; Drommelschmidt et al., 2017).

#### Endothelial Cell

**FIGURE 5:**
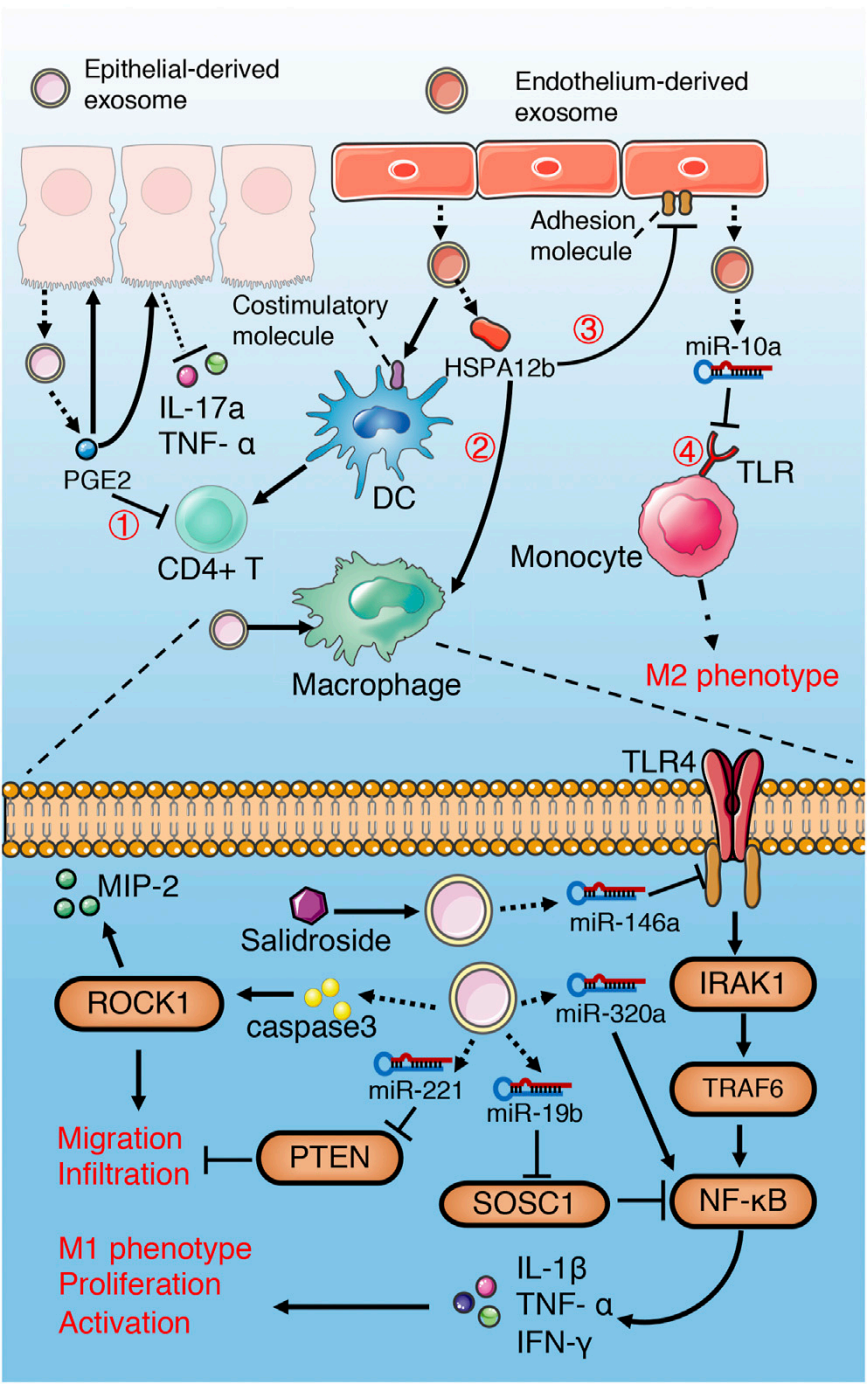
Role of exosomes derived from epithelial and endothelial cells in sepsis. Epithelium-derived exosomes promote activation, proliferation, migration, infiltration and M1 polarization of macrophages through multiple miRNAs-mediated NF-κB activation, PTEN inhibition and caspase3-mediated ROCK1 activation. However, salidroside can induce epithelial exosomes releasing miR-146a, which inhibit TLR4/IRAK1/TRAF6/NF-κB signaling pathway. Exosomal PGE2 of IEC inhibit T cells through cAMP/PKA-dependent pathway^①^, and decrease pro-inflammatory cytokines release *via* autocrine and paracrine. Exosomal HSPA12b of endothelium reduce pro-inflammatory cytokines release and adhesion of macrophages by Inhibiting NF-κB^②^ and activating PI3K/Akt^③^ signaling separately. Endothelial exosomes can induce a M2-like phenotype in monocyte through Inhibiting TLR/IRAK4/TAK1/NF-κB^④^ signaling pathway and promote DC maturation and T cell activation.

Heat shock protein A12B (HSPA12B) is mainly expressed in endothelial cells and transferred from released exosomes to macrophages (Figure 5). By inhibiting the activation and nuclear translocation of NF-kB, it significantly increases the IL-10 level of LPS-stimulated macrophages and reduces the production of TNF-α and IL-1β (Tu et al., 2020). In addition, HSPA12B reduces the expression of LPS-induced adhesion molecules and the production of pro-inflammatory cytokines *via* activating the PI3K/Akt signaling pathway of target cells (Li et al., 2013), which contribute to reduce the migration and adhesion of macrophages to target cells in sepsis (Tu et al., 2020). Endothelial microparticles (EMP) has been shown to promote the maturation of plasma cell-derived dendritic cell (PDC) by up-regulating the expression of costimulatory molecules and promoting the release of IL-6 and IL-8 (Angelot et al., 2009). PDC is considered to be the main cell secreting IFN- α under bacterial stimulation (Gilliet et al., 2008), and can induce the activation of prime T cells (Angelot et al., 2009). EVs (including exosomes) released by unstimulated endothelial cells are rich in a variety of miRNA (Njock et al., 2015). miR-10a in exosomes inhibits NF-κB by targeting suppression of IRAK4, TAK1/MAP3K7, and β-TRC, while miR-12b and miR-181b reduce the expression of pro-inflammatory genes and increase the expression of immuno-modulatory genes of monocytes *via* inhibiting the nuclear translocation of NF-κB and IRF5, and promote the differentiation of monocytes to anti-inflammatory M2-like phenotype (Njock et al., 2015). The regulation of endothelial EVs (including exosomes) on monocyte differentiation is stimulus dependent. EVs secreted by human umbilical vein endothelial cells stimulated by OX-LDL induce M1-like phenotype *via* miR-155, while EVs stimulated by KLF2 lead to M2-like phenotype in monocytes (He et al., 2018). These findings indicate that exosomes derived from endothelial cells have multiple effects and may play a role in balancing immunity in sepsis.

#### Epithelial Cell

The effect of lung epithelium-derived exosomes on macrophages in sepsis is mainly characterized by immune activation and pro-inflammation (Figure 5). Hyperoxia-induced, lung epithelial cell-derived and caspase-3 enriched EVs (including exosomes) activate macrophage *via* the ROCK1 pathway, and increase secretion of pro-inflammatory cytokines and macrophage inflammatory protein 2 (MIP-2) (Moon et al., 2015). Similarly, hyperoxia increase the levels of miR-320a and miR-221 in EVs derived from epithelial cells, and promote the activation and recruitment of macrophages and the release of pro-inflammatory cytokines by activating MMP9 and NF-κB (Lee et al., 2016). EVs derived from acid-induced lung epithelial cell are rich in miR-17 and miR-221, which increase macrophage infiltration through promoting integrin β1 circulation, inhibiting PTEN and activating c-fos-Rab11 signaling pathways (Lee et al., 2017). However, salidroside can upregulated the expression of miR-146a in LPS-induced pulmonary epithelial cell-derived exosomes, which reduce the pro-inflammatory cytokines release of macrophages *via* inhibiting TLR4/IRAK1/TRAF6/NF-κB signaling pathway (Zheng et al., 2020).

The intestinal epithelial cell (IEC)-derived luminal EVs during sepsis inhibit the release of pro-inflammatory cytokines TNF- α and IL-17a through autocrine and paracrine (Appiah et al., 2020). Exosomal PGE2 of IEC inhibit the activation of NKT and CD4+ T cells through cAMP/PKA-dependent pathway (Deng et al., 2013). In addition, IEC-derived pro-inflammatory cytokine enriched exosomes promote the migration of macrophages and aggravate the inflammatory response (Mitsuhashi et al., 2016). Exosomal miRNA-19b-3p of tubular epithelial cells (TEC) promotes M1 macrophage activation *via* SOCS-1 inhibition and NF- κB activation (Lv et al., 2019). Choroid plexus epithelial cells (CPE) release EVs containing miR-146a and miR-155 into cerebrospinal fluid (CSF) during sepsis, which trigger target mRNA repression in glial cells and induce an inflammatory response (Balusu et al., 2016).

#### Fibroblasts

The exosomes produced by synovial fibroblasts from patients with rheumatoid arthritis (RASF) contain membrane bound forms of TNF- α, which contributes to T cells apoptosis resistance *via* AKT and NF-kB activation (Zhang et al., 2006). According to the result, we may infer that exosomes derived from fibroblasts may play a similar role and alleviate immunosuppression in the later phase of sepsis, which need further study.

### Immunomodulatory Effect of Exosomes Derived From Immune Cells in Sepsis

#### Macrophage

**FIGURE 6:**
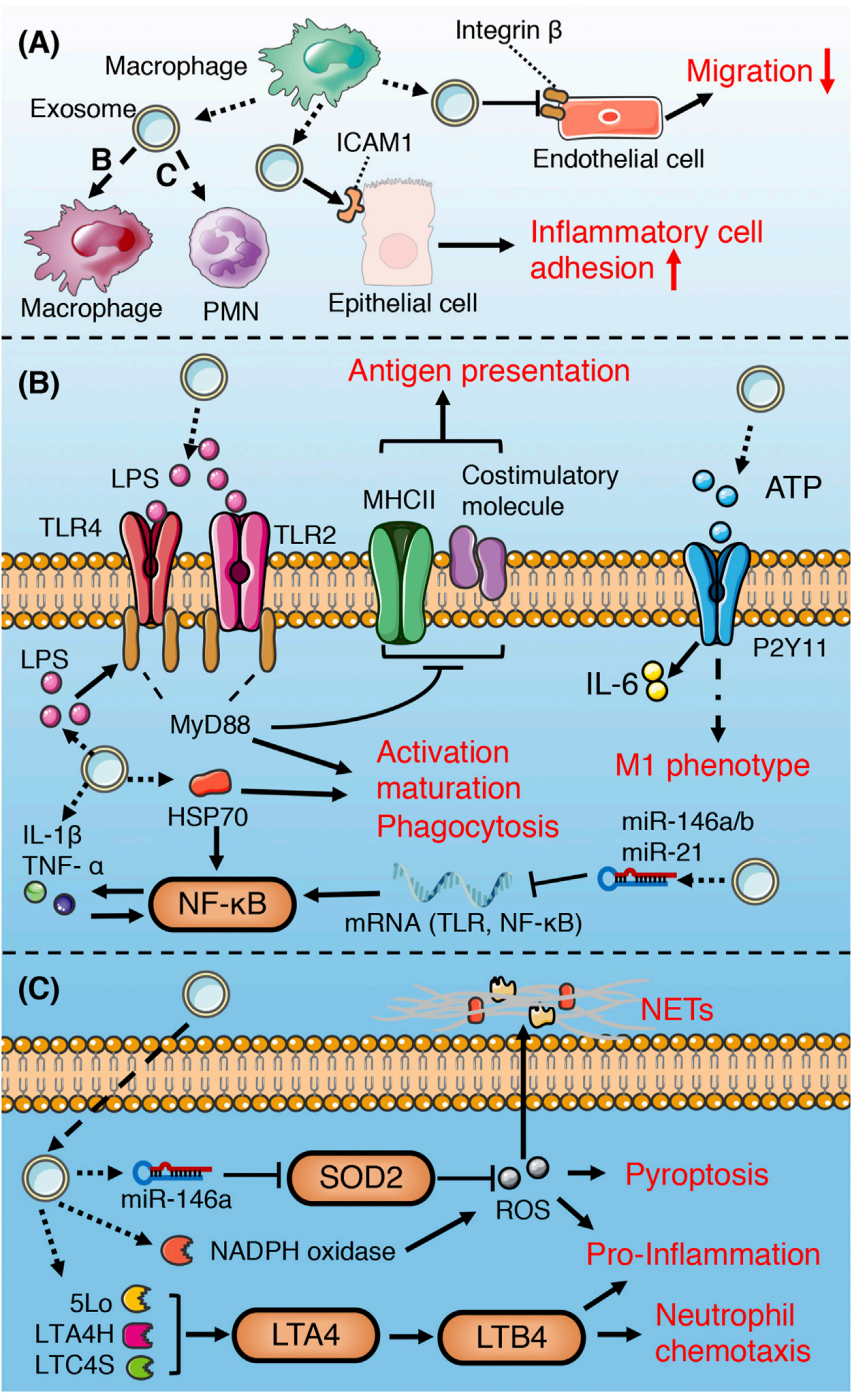
Role of macrophage-derived exosomes in sepsis **(A)** macrophage-derived exosomes can induce pro-inflammatory response in recipient macrophage and PMN, promote inflammatory cell adhesion to endothelium, and suppress migration of epithelial cells **(B)** macrophage-derived exosomes containing DAMPs and PAMPs promote recipient macrophage activation, maturation, phagocytosis, and M1 polarization, while suppress the ability of antigen presentation. **(C)** Exosomal enzymes and miRNA increase NETs formation, chemotaxis, pro-inflammatory response, and pyroptosis of PMN.

As an important part of the innate immune system, macrophages are the first line of defense against pathogen invasion. A number of studies have shown that exosomes derived from activated macrophages can affect the immune function of inactivated macrophages through autocrine and paracrine (Bhatnagar et al., 2007; Neyrolles et al., 2010; Ojcius et al., 2011; Sakaki et al., 2013; McDonald et al., 2014; Li et al., 2018b; Nair et al., 2018) (Table 2) (Figure 6). In addition, exosomes derived from macrophages also affect neutrophils (Esser et al., 2010; Jiao et al., 2017; Zhang et al., 2019b), alveolar epithelial cells (Soni et al., 2016), endothelial cells (Lee et al., 2014) and hepatocytes (Wang et al., 2018a) in sepsis (Table 2) (Figure 6).

**TABLE 2:** Immunomodulatory effect of exosomes derived from immune cells in sepsis.

Donor cell	Contents	Transfer pathway	Target cell	Signaling pathway/Protein	Immuno-effect	Immune outcome	References
Macrophage	19KD lipoprotein	N/A	Macrophage	TLR2-MyD88 (+), CIITA (−)	MHC-II ↓, CD64 ↓	Suppression	Ojcius et al. (2011)
Macrophage	HSP70	N/A	Macrophage	NF-κB (+)	Macrophage mutation and phagocytosis ↑, TNF-α ↑	Activation	Neyrolles et al. (2010)
Macrophage, THP1	PAMPs (19KD lipoprotein, LAM, LPS)	N/A	Macrophage	TLR2/4-MyD88 (+)	Macrophage and neutrophil recruitment ↑, macrophage activation ↑, TNF-1/α ↑, IL-12 ↑	Activation	Bhatnagar et al. (2007)
Macrophage	miR-146a	N/A	Neutrophil	SOD (−)	ROS ↑, NETs ↑	Activation	Zhang et al. (2019b)
Alveolar macrophage	MV^a^: TNF	N/A	Alveolar epithelial cell	N/A	ICAM-1 ↑	Activation	Soni et al. (2016)
Macrophage	N/A	N/A	Macrophage	NF-κB (+)	TNF-α ↑	Activation	Li et al. (2018b)
Macrophage	N/A	N/A	PMN	NADPH oxidase (+)	ROS ↑, pyroptosis ↑	Activation	Jiao et al. (2017)
Macrophage	N/A	N/A	HUVEC	Integrin β1 ubiquitin, internalization and degradation (+), integrin β1 recycle to endosome (−), MEK-ERK (−)	MMP9 ↓, ECs migration ↓	N/A	Lee et al. (2014)
Macrophage	N/A	N/A	Hepatocyte	NOD (+)	NLRP3 ↑	Activation	Wang et al. (2018a)
Macrophage	MV^a^: Histone	N/A	Naive macrophage	TLR4 (+)	TNF-α ↑, IL-6 ↑, IL-1β ↑	Activation	Nair et al. (2018)
Macrophage	miR-126-5p, miR-146a/b, miR-21-3p, let7b, mRNA (encoding GADPH, CXCL2, CCL2/4, TNF-α), Ieb2, creb, G-CSF, IL-1Ra, TNF-α, chemokines	N/A	Macrophage	TLR-NF-κB	Anti-inflammation, pro-inflammation	Suppression/Activation	McDonald et al. (2014)
MDM, MDDC, PMNL	LTC4S, LTA4H, 5-LO	TGF-β1	PMNL	5-KETE (+), LTA4-LTB4-LTC4 (+)	PMNL chemotaxis ↑, inflammatory cytokines ↑	Activation	Esser et al. (2010)
Macrophage	IL-1β, NLRP3	ATP/P2X7R	N/A	NF-κB (+)	Inflammatory cytokines ↑	Activation	Qu et al. (2007)
Monocyte	MP^a^: mt DNA	N/A	PMNs	TLR9 (+)	PMNs chemotaxis ↓	Suppression	Konecna et al. (2021)
Monocyte	N/A	N/A	Monocyte	TLR4 (−)	TNF-α ↓	Suppression	Wisler et al. (2020)
Monocyte	miR-155, miR-223	N/A	ECs (direct) PMNs (indirect)	TLR4-NF-κB (+)	ICAM-1 ↑, CCL-2 ↑, IL-6 ↑, monocyte chemotaxis and adhesion ↑	Activation	Tang et al. (2016)
Monocyte (THP1)	ATP	SLC179A	THP1	P2Y11 (+)	M1 phenotype ↑, IL-6 ↑	Activation	Sakaki et al. (2013)
PMN	MP^a^: AnxA1	N/A	PMNs	ALX (bind to AnxA1)	PMNs chemotaxis and adhesion ↓	Suppression	Dalli et al. (2008)
PMN	MP^a^: Anti-inflammatory cytokines (late stage in sepsis), PS	N/A	THP1 Macrophage	N/A	THP1 (MP): TGF-α ↑, PGE2 ↑, IL-10 ↑, THP1 (bystander): activity ↓, M2 ↑, M1 ↓	Suppression	Prakash et al. (2012)
PMN	PS	N/A	IMoDC	TLR4 (+)	iMoDC maturation, phagocytosis and chemotaxis ↓, induce T cell proliferation ↓, TGF-β1 ↑, CCR7 ↓	Suppression	Eken et al. (2008)
IDC (immature dendritic cell)	MFGE8	N/A	Macrophage	N/A	Phagocytosis ↑ (direct), TNF-α ↓, HMGB1 ↓ (indirect)	Activation (direct) Suppression (indirect)	Miksa et al. (2009)
BMDC	EVs^a^: TLR4	N/A	BMDC	NF-κB (+)	IL-6 ↑, TNF-α ↑	Activation	Zhang et al. (2019a)
DC	ICAM-1, MHC-II-peptide, CD86, MFG-E8	ICAM-1/LFA-1, ICAM-1/Mac-1	Naive T cell B cell Macrophage	ICAM-1/LFA-1 (+), ICAM-1/Mac-1 (+), MHC-II-peptide/TCR (+)	Naive T cell activation ↑, T Cell proliferation ↑, Transfer the ability of priming naive T cells to B cell and macrophage	Activation	Segura et al. (2005)
IDC	MFGE8	N/A	Macrophage	αVβ3- MFGE8-PS (+)	Phagocytosis ↑, IL-6 ↓, TNF-α ↓	Suppression	Miksa et al. (2006)
DC	miR-155, miR-146a	N/A	DC	miR-155: AGO-BACH1 (−), AGO-SHIP1 (−), miR-146a: IRAK1 (−), TRAF6 (−)	miR-155: IL-6 ↑, miR-146a: IL-10 ↑, IL-6↓, IL-12p40 ↓	Activation/Suppression	Alexander et al. (2015)
DC (LMoDC)	EVs: HLA-1, ICAM-1, miR-155, CD63	N/A	CD8+ T cell	MHC-peptide complex	CD8^+^ T cell activation ↑, TNF ↑, IFN ↑	Activation	Lindenbergh et al. (2019)
Treg	EVs^a^: miR-142-3p, miR-150-5p	N/A	DC	ICAM-1/LFA-1 (+)	IL-6 ↓, IL-10 ↑, MHC-II ↓, CD80 ↓	Suppression	Tung et al. (2018)
Treg	miR-155, let-7b, let-7d	Rab27, ceramide	Th1 cell	COX2 (+)	IFN-γ ↓, T cell proliferation ↓	Suppression	Okoye et al. (2014)
B cell	miR-155 inhibitor	N/A	Macrophage	N/A	TNF-α↓, SOCS1 mRNA↑	Suppression	Momen-Heravi et al. (2014)
B cell	MHC-peptide complex	Igα/β, TCR	CD4+ T cell	MHC-peptide-TCR (+)	CD4^+^ T cell activation ↑, antigen specific memory T cells ↑	Activation	Muntasell et al. (2007)
CD8+T cell	TCR, FasL	TCR-MHC-I CD54-LFA-1	DC (direct) CD8+T cell (indirect)	TCR-MHC-I (−) FasL-FasL (+)	DC antigen presentation ↓, apoptosis ↑, CD8^+^ CTL ↓	Suppression	Xie et al. (2010)
T cell	MP^a^: Monocyte activating factors	N/A	Monocyte/macrophage	N/A	TNF-α ↑, IL-1 ↑, SIL-Ira ↑	Activation	Scanu et al. (2008)
MDSC (inhibitory phenotype)	lncRNA (Hotairm1)	N/A	MDSC (activation phenotype)	S100A9 nuclear translocation +＋)	S100A9 release ↓, pro-inflammatory cytokines ↓, MDSCs transfer to inhibitory phenotype	Suppression	Alkhateeb et al. (2020)
G-MDSC	N/A	N/A	Neutrophil Monocyte/macrophage	L-arginine metabolism (−), ROS (−)	Neutrophil and monocyte/macrophage infiltration ↓, Th1 proliferation ↓, Treg ↑, IFN-γ ↓, TNF-α ↓	Suppression	Wang et al. (2016)

^a^ Exosomes are subsets of EVs or MVs, however, their immunomodulatory effects may exist unpredictable differences.

Pathogen-associated molecular patterns (PAMPs) can stimulate macrophages to release ATP, while extracellular ATP, as a damage-associated molecular patterns (DAMPs), can activate the innate immune system and promote the production of inflammasome (Sutterwala et al., 2006; Piccini et al., 2008). Hayato et al. found that ATP enriched exosomes derived from LPS-challenged macrophages can promote recipient macrophage activation *via* activating P2Y11 receptors (Sakaki et al., 2013). Similarly, several studies have illustrated that PAMPs (such as HSP70, LAM, LPS)-containing exosomes released by pathogen-infected macrophages promote the activation, maturation and phagocytosis of uninfected macrophages *via* activation of TLR/MyD88/NF-KB signaling pathway (Bhatnagar et al., 2007; Neyrolles et al., 2010; Nair et al., 2018). Therefore, the extracellular release of exosomes containing PAMPs may be one of the important mechanisms of immune surveillance. This exosome-mediated autocrine or paracrine pathway accelerates immune activation triggered by PAMPs. However, exosomes derived from macrophages infected with Mycobacterium tuberculosis inhibit the expression of MHC II and CD64 molecules on the surface of naïve macrophages in a TLR2-MyD88-dependent manner, and reduce their ability of antigen presentation (Ojcius et al., 2011). The effect of exosomes on the immune response is dynamic and multifactorial. Exosomes released from LPS-challenged macrophages can both promote and inhibit aspects of immunity and inflammation. For instance, macrophages stimulated with LPS secrete exosomes containing elevated levels of cytokines and miRNAs, some of which display the opposite effects (McDonald et al., 2014). MiR-21-3p, miR-146a and miR-146b in exosomes prevent over-activation of innate immune system by inhibiting TLR/NF-κB signaling pathway (Taganov et al., 2006; Hsu et al., 2011; McDonald et al., 2014), while pro-inflammatory cytokines and chemokines in exosomes lead to activation of NF- κB and promote inflammatory response and innate immune cell chemotaxis (McDonald et al., 2014; Kovach et al., 2016).

Exosomes secreted by human macrophages contain enzymes for leukotriene biosynthesis (LTC4S, LTA4H, 5-LO) and promote granulocyte migration (Esser et al., 2010). miR-146a enriched exosomes derived from oxLDL-stimulated macrophages induce ROS production and NETs formation *via* targeted inhibition of SOD2 (Zhang et al., 2019b). Exosomes released from macrophages induced by hemorrhagic shock increase the ROS production and pyroptosis of neutrophils by activating NADPH oxidase (Jiao et al., 2017), which may be involved in the immunosuppression during sepsis.

Macrophage-derived exosomes promote the adhesion of inflammatory cells in sepsis by up-regulating the expression of ICAM-1 in alveolar epithelial cells (Soni et al., 2016). In addition, exosomes secreted by macrophages suppress endothelial cell migration through regulating integrin trafficking (Lee et al., 2014). Therefore, the exosomes released by macrophages may aggravate physiological barrier dysfunction and organ damage induced by sepsis.

In conclusion, the macrophage-derived exosomes in sepsis mainly promote immune activation and mediate pro-inflammatory response and tissue damage.

#### Monocyte

**FIGURE 7:**
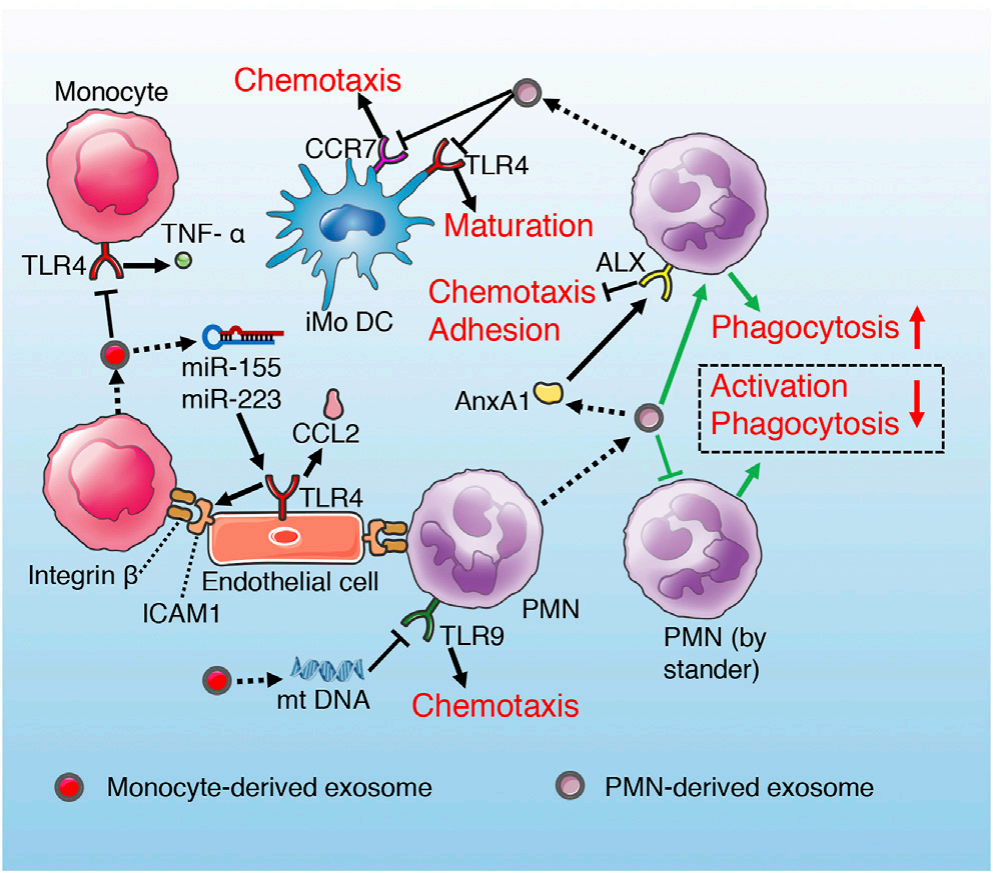
Role of exosomes derived from monocyte and PMN in sepsis. Monocyte exosomes reduce TNF-α release of recipient monocyte and PMN chemotaxis *via* TLR inhibition. However, exosomal miRNAs of monocyte promote endothelium adhesion by activating TLR. PMN-derived exosomes suppress maturation and chemotaxis of iMoDC through inhibiting CCR7 and TLR4 separately. PMN-derived exosomes suppress recipient PMN chemotaxis and adhesion to endothelium *via* AnxA1/ALX pathway. In addition, exosomes secrete by PMN promote anti-inflammatory cytokines release of recipient PMN, while induce immune anergy of bystander PMN (green routes).

Monocytes stimulated by Inflammation and infection release exosomes containing mitochondrial damage-associated molecular patterns (mt-DAMPs), which reduce the chemotaxis and sterilization of neutrophils through TLR9 inhibition mediated by endosomal acidification (Itagaki et al., 2011; Konecna et al., 2021). LPS can stimulate endothelial cells to express adhesion molecule ICAM-1, which can bind to monocytes and trigger inflammatory response (Wang et al., 2015a). In addition, miR-155 and miR-223, in monocyte-derived exosomes stimulated by LPS upregulate the expression of ICAM-1, chemokine ligand (CCL)-2 and cytokine IL-6 by activating TLR4/NF-KB signaling pathway in endothelial cells (Tang et al., 2016), which further aggravates inflammatory response and endothelial injury through positive feedback loop (Figure 7). Thus, we can infer that monocyte-derived exosomes not only promote the aggregation of monocytes to endothelial cells to cope with PAMPs, but also control excessive inflammatory response by inhibiting the chemotaxis of neutrophils. In addition, proteomic analysis demonstrated that exosomes derived from LPS-stimulated monocytes contain protein networks with potential immunosuppressive patterns (Wisler et al., 2020). These exosomes reduce LPS-induced TNF-α release from recipient monocytes (Wisler et al., 2020), which suggests a exosomal negative feedback mode of monocyte limiting self-overactivation (Figure 7).

#### Neutrophils/Polymorphonuclear Leukocytes

The exosomes derived from polymorphonuclear leukocytes (PMNs) contain functional annexin 1 (AnxA1), which is an endogenous anti-inflammatory protein by controlling activation and trafficking of the inflammatory cells (Dalli et al., 2008; Senchenkova et al., 2019). AnxA1 can activate and bind ALX receptors in PMNs, and inhibit the chemotaxis, adhesion and migration of PMNs to endothelial cells (Chiang et al., 2006; Hayhoe et al., 2006; Senchenkova et al., 2019). It has been demonstrated that AnAX1-rich exosomes derived from PMNs suppress recipient PMN migration and adhesion to human umbilical vein endothelial cell (HUVEC), which is a manner of self-regulation alleviating inflammatory response and endothelial injury induced by excessive PMNs aggregation (Dalli et al., 2008). Similar study also supports the anti-inflammatory effects of exosomes derived from neutrophils (Prakash et al., 2012). However, this anti-inflammatory effect may aggravate immunosuppression in the late stage of sepsis. PMN-derived exosomes lead to increased activation and enhanced phagocytosis and increased secretion of antimicrobial factors (TGF-α, PGE2, IL-10) of the recipient PMN, while result in immune anergy of the by stander PMN characterized by deactivation and decreased phagocytosis (Prakash et al., 2012) (Figure 7). The immunosuppressive effect of PMN-derived exosomes is also reflected in the hindrance of DCs maturation (Figure 7). Ceylan et al. (Eken et al., 2008) found that extracellular vesicles (including exosomes) derived from PMN interfere with the maturation of immature monocyte-derived dendritic cell (iMoDC) induced by LPS, decrease its phagocytosis and chemotaxis, and weaken its capacity to promote T cell activation and proliferation (Figure 7). Phosphatidylserine (PS) in PMN-derived exosomes has been identified as a major factor influencing iMoDC maturation and function (Eken et al., 2008; Ohyagi et al., 2013). In addition, iMoDC exposed to PMN-derived exosomes release TGF-β1, which is responsible for the downregulation of TLR4-mediated maturation of iMoDC and CCR7-mediated chemotaxis of DCs (Eken et al., 2008; Pang et al., 2016; Wang et al., 2018b).

#### Dendritic Cell

Dendritic cells (DCs) are the professional antigen presenting cell (APCs), which can efficiently uptake, process and present antigens. Immature DC (IDC) has stronger phagocytosis and migration ability, while mature DC expresses higher levels of adhesion and co-stimulatory molecules, which can effectively activate prime T cells and play a central role in initiating, regulating and maintaining immune response (Miksa et al., 2006; Yang et al., 2014). It has been demonstrated that DC-derived exosomes contain MFGE8, CD63, Toll-like receptors, adhesion molecules, co-stimulatory molecules, MHC-peptide complexes, and miRNA, which regulate the immune function of DCs, macrophages and lymphocytes during sepsis (Segura et al., 2005; Miksa et al., 2006; Miksa et al., 2009; Alexander et al., 2015; Zhang et al., 2019a; Lindenbergh et al., 2019).

**FIGURE 8:**
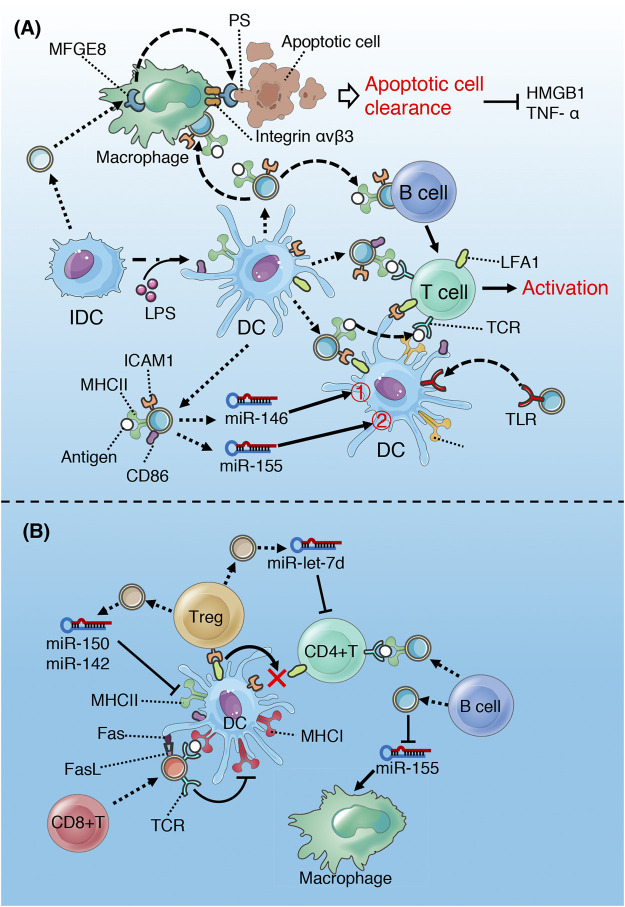
Role of exosomes derived from DC and lymphocyte in sepsis. **(A)** IDC-derived exosomes promote MFGE8-mediated phagocytosis of apoptotic cells by macrophage. DC-derived exosomes can activate antigen-specific T cells according to antigen presentation described above. In addition, miR-146 in DC-derived exosomes alleviate inflammatory response of recipient DC *via* inhibiting IRAK1/TRAF6/NF-κB^①^, while exosomal miR-155 aggravate inflammatory response by suppressing BACH1 and SHIP1^②^ signaling pathways. **(B)** exosomes derived from Treg induce an immune tolerance phenotype of DC and inhibit T cell proliferation. Exosomes released by CD8+ T cell inhibit the ability of DC to activate T cells and induce DC apoptosis. However, exosomes derived B cell can activate T cell, and also be used as drug carrier of immunotherapy.

The exosomes released by IDCs contain MFGE8 (Miksa et al., 2006; Miksa et al., 2009), which is a required protein for the opsonization of apoptotic cells for phagocytosis (Hanayama et al., 2004). The integrin α V β 3 expressed on the surface of macrophages binds to the exposed PS on the surface of apoptotic cells through the mediation of MFGE8 to complete the phagocytosis process (Miksa et al., 2006). The decrease of MFGE8 contributes to the accumulation of apoptotic cells, leading to a surge in pro-inflammatory cytokines, which is responsible for the progression and deterioration of sepsis (Shah et al., 2012). The IDC-derived exosomes promote the clearance of apoptotic cells *via* transferring MFGE8 to macrophages, which in turn reduce the release of TNF-α and HMGB1 (Miksa et al., 2006; Miksa et al., 2009) (Figure 8A). Compared with exosomes from IDC, LPS-stimulated mature DC-derived exosomes contain more MHC-II-peptide complexes, ICAM-1 and CD86, which can activate antigen-specific T cells more effectively (Segura et al., 2005). DC-derived exosomes perform antigen presentation and activate T cells through the pathways described above (Figure 2). It is noteworthy that the ability of DC-released exosomes to activate naive T cells may also be magnified by activated bystander T cells (Lindenbergh et al., 2019). After migration to the lymph node, the gradually matured DC stimulated by LPS interact with activated bystander T cells, which promote the further maturation of DC and subsequent release of exosomes containing more HLA-1, ICAM-1, miR-155, and CD63 (Lindenbergh et al., 2019).

It has been found that EVs (exosomes) can mediate the transmission of TLRs between DCs, which may improve the reactivity of DC to LPS and accelerate the activation of immune defense and inflammatory response (Zhang et al., 2019a). In addition, LPS-stimulated DC-derived exosomes are enrich in miR-155 and miR-146a, which can modulate the inflammatory response of recipient DC through paracrine (Alexander et al., 2015) (Figure 8A). Exosomal miR-155 reduces the expression of anti-inflammatory target genes BACH1 and SHIP1, while exosomal miR-146a reduces the expression of pro-inflammatory target genes IRAK1 and TRAF6 (Alexander et al., 2015). The inflammatory response of recipient cells is buffered by the two exosomal microRNAs with opposite functions, so as to achieve the best response amplitude. One hypothesis may be that miR-155 and miR-146a exist in different exosomes or be released at different phase during the dynamic process of the disease to balance immune status and inflammatory response, which needs to be confirmed by further studies.

#### Lymphocyte

##### T Lymphocyte

It has been demonstrated that molecular transfer between DCs and T cells is bidirectional. Similar to DCs, active T cells can also release bioactive exosomes, which can be recruited by APCs or B cells (Xie et al., 2010). Xie et al. found that TCR-carrying exosomes released by activated CD8 + T cells could weak the ability of DCs to activate other antigen-specific T cells (Xie et al., 2010). On the one hand, the exosomes down-regulate the expression of MHC-I in DC *via* TCR-mediated internalization, on the other hand, the TCR released by the exosomes compete with antigen-specific T cells to bind to the MHC-I-peptide complex on the surface of DC, thus interfere with T cell activation (Xie et al., 2010). In addition, exosomal FasL induces DC apoptosis through Fas/FasL signaling pathway (Xie et al., 2010). Therefore, activated T cell-derived exosomes inhibit immune over-activation through negative feedback mechanism (Figure 8B).

Regulatory T cells (Tregs) are a subset of T cells, which can maintain self-tolerance and limit other immune responses. The over-activation of Tregs is considered to be an important cause of immunosuppression in late sepsis. It has been found that DCs and Th1 cells are the main targets for the immuno-modulatory function of Treg and its derived exosomes (Okoye et al., 2014; Tung et al., 2018) (Figure 8B). MiR-150-5p and miR-142-3p in Treg-derived exosomes induce the immune tolerance phenotype of DC by down-regulating the expression of surface molecules MHC-II and CD80 (Tung et al., 2018). Similarly, let-7d in Treg-derived exosomes inhibits IFN-γ release and Th1 cell proliferation through COX2-dependent pathway (Okoye et al., 2014). It is worth noting that the immune synapse (IS) formed between Treg and DC can enhance the functional transmission of exosomes to recipient DC (Gutierrez-Vazquez et al., 2013; Tung et al., 2018). However, the consumption of adhesion molecules (such as Fascin-1) to build IS between Treg and DC can reduce the efficiency of IS formation between DC and effector T cells, thus affecting the antigen presentation and activation of effector T cells by DC and its exosomes, which may aggravate immunosuppression to some extent (Tung et al., 2018) (Figure 8B).

##### B Lymphocyte

It has been found that engagement of antigen-loaded B cells with specific CD4 T cells can trigger exosomes release form B cells (Muntasell et al., 2007). The MHC-II-peptide complexes carried by the exosomes can effectively activate CD4+T cells (Muntasell et al., 2007) (Figure 8B). Although B cells can not play a major role in the early initiation of naive T cells compared with DCs, the exosomes secreted by B cells can act as a modulator of continuous immune response or play a role in maintaining antigen-specific memory T cells (Muntasell et al., 2007). In addition, exosomes derived from B cells can also be used as carriers to transport miR-155 inhibitor to macrophages, reducing the TNF- α release through SOCS1/NF-KB-dependent pathway (Momen-Heravi et al., 2014).

##### Natural Killer Cell

As an important cell of the immune system, Natural killer (NK) cells play the role of non-specific target cell killing and immune regulation. The research on NK cells and their exosomes mainly focuses on the field of anti-tumor and anti-virus, but little is known about the role of NK cells in sepsis. It has been found that the exosomes derived from NK cells is structural and independent on the activation of donor cells, which only has an effect on activated recipient immune cells, suggesting a role in immune surveillance and homeostasis in sepsis (Lugini et al., 2012).

##### Myeloid-Derived Suppressor Cell

Myeloid-derived suppressor cells (MDSCs) is a group of heterogeneous cells derived from bone marrow, which are the precursor of DCs, macrophages and granulocytes and have significant inhibitory effect on immune cell response. It has been demonstrated that, MDSCs play different roles in the modulation of inflammation and immune response with the development of sepsis (Brudecki et al., 2012; Dai et al., 2017). MDSCs produced in acute/early sepsis is pro-inflammatory phenotype (Brudecki et al., 2012), while in chronic/late sepsis is immunosuppressive phenotype (Dai et al., 2017). In late sepsis, MDSCs-derived exosomes contain high levels of LncRNA Hotairm1, which can transform MDSCs from pro-inflammatory phenotype to immunosuppressive phenotype by promoting nuclear translocation of pro-inflammatory protein S100A9 (Alkhateeb et al., 2020). Exosomes released by granulocytic MDSCs (G-MDSCs) can attenuate the inflammatory response of mice with colitis induced by DSS, reduce the infiltration of innate immune cells, suppress the proliferation of Th1 cells and promote the activation of Treg cells (Wang et al., 2016). This inhibitory effect on innate and adaptive immunity may be achieved through the inhibition of L-arginine metabolism and ROS (Zea et al., 2005; Tian et al., 2015).

## The Therapeutic Use of Exosomes in Sepsis

Exosomes are considered to be natural nanoliposomes, which are relatively stable in the circulation, and have the ability to resist complement lysis and ribonuclease attack (Chaput and Théry, 2010). In addition, exosomes are low in immunogenicity and well tolerated (Wu et al., 2017). These characteristics ensure exosomes to be excellent therapeutic vehicles.

MSCs-derived exosomes are most used in the treatment of sepsis. Exosomes isolated from MSCs treated by LPS *in vitro* can stimulate the regenerative and reparative properties of the target cells (Wu et al., 2017). Intravenous injection of miR-223 enriched exosomes derived from MSCs can reduce myocardial injury in sepsis through inhibiting the release of pro-inflammatory cytokines from macrophages (Wang et al., 2015c). EVs released from MSCs can reduce pulmonary edema induced by LPS and alleviated inflammation (Zhu et al., 2014). In addition, MSC-derived EVs have been shown to reduce LPS-induced motor neuron inflammation and brain injury of septic rats (Rajan et al., 2016; Drommelschmidt et al., 2017).

Exosomes isolated from the peritoneum and bronchoalveolar lavage fluid of patients with surgical sepsis can significantly enhance the activity and phagocytic capacity of THP-1 monocytes *in vitro* (Prakash et al., 2012). Exogenous administration of exosomes containing MFG-E8 from DC or IDC can accelerate the clearance of apoptotic cells accumulated in sepsis (Miksa et al., 2009). Studies have shown that exosomes isolated from B cells can be used as ideal carriers of synthetic miRNA inhibitors to reduce the release of macrophage pro-inflammatory factors in sepsis (Momen-Heravi et al., 2014). Injection of curcumin-containing exosomes can reduce the inflammatory response in septic rats and increase the survival rate (Sun et al., 2010). Similarly, curcumin-containing exosomes can pass through the blood-brain barrier when administered intranasally, and effectively reduce brain inflammation caused by LPS (Zhuang et al., 2011). In addition, tumor-derived exosomes are used to reduce excessive inflammation in sepsis by virtue of their immunosuppressive properties. It has been demonstrated that exosomes produced by H22 hepatic tumor cells can protect mice from severe tissue injury induced by LPS (Teng et al., 2012). However, the potential tumorigenicity of these exosomes should be taken into consideration more carefully.

## Conclusion and Perspectives

The role of exosomes in sepsis is multiple and complex. The exosomes derived from antigen-presenting cells (DCs, macrophages and B cells) mainly contribute to immune activation, including promoting the activation, differentiation, maturation and proliferation of immune cells, which assist the innate and adaptive immune system to respond to the invasion of pathogens more efficiency. On the contrary, MSC and Treg derived exosomes mainly exert immunosuppressive and anti-inflammatory effects. However, exosomes from other cell sources show two sides. Especially exosomes derived from activated inflammatory cells (monocytes and neutrophils) may inhibit their over-activation through autocrine or paracrine-mediated negative feedback mechanism, while may also accelerate the recruitment to the inflammatory site through the positive feedback pathway. This bi-directional and self-limited regulation of exosomes play an important role in maintaining the homeostasis of sepsis, which may act as the regulator or balance switch of the immune system.

It is worth noting that exosomes from the same source have different regulatory effects on the immune system in sepsis, which may be due to the following reasons: 1) the same origin exosomes contain a variety of contents with different bioactivities. 2) the effects of exosomes *in vivo* and *in vitro* may be different. 3) sepsis is a dynamic and complex pathological process, and the same origin exosomes in the early stage (excessive inflammatory response) and the late stage of sepsis (immunosuppression) may have opposite effects.

In conclusion, the complex role of exosomes in sepsis is related to multiple factors involving cell sources, contents and phase of disease. Endogenous exosomes can not only aggravate the inflammatory response and organ injury of sepsis, but also play a protective role by balancing immunity. While the engineered exogenous exosomes are used for immunotherapy in different stages of sepsis according to their immune characteristics or as a drug carrier. Understanding the specific immunomodulatory characteristics and mechanism of exosomes from various cell sources in sepsis is helpful for us to choose the right time and target, seeking benefits and avoiding disadvantages in the treatment of sepsis. As an immunomodulatory switch, exosomes may bring a new dawn for the treatment of sepsis in the future.
